# Division of an Iliac Crest Bone Biopsy Specimen to Allow Histomorphometry, Immunohistochemical, Molecular Analysis, and Tissue Banking: Technical Aspect and Applications

**DOI:** 10.1002/jbm4.10424

**Published:** 2020-10-29

**Authors:** Sylvain Picard, Christian N Mayemba, Roth‐Visal Ung, Simon Martel, Fabrice Mac‐Way

**Affiliations:** ^1^ Centre Hospitalier Universitaire (CHU) de Québec Research Center, L'Hôtel‐Dieu de Québec Hospital, Endocrinology and Nephrology Axis, Faculty and Department of Medicine Université Laval Quebec City Quebec Canada

**Keywords:** BONE HISTOMORPHOMETRY, ILIAC CREST BIOPSY, METABOLIC BONE DISEASES, MOLECULAR BIOLOGY, TISSUE BANKING

## Abstract

The evaluation of bone complications in chronic kidney disease (CKD) often requires a bone biopsy, the analysis of which can refine the diagnosis of bone defects. Bone histomorphometry performed on sections of the iliac crest biopsy remains the reference procedure for the quantitative assessment of bone health in CKD patients, whereas immunohistochemistry and other molecular biology analyses are indispensable tools for studying the disrupted signaling pathways. Traditionally, the whole iliac crest biopsy was included in methyl‐methacrylate (MMA) and was exclusively used for bone histomorphometry to describe static, dynamic, and structural parameters. Therefore, further molecular analysis of the bone tissue or the need for tissue banking would require a second biopsy to be made, because inclusion in MMA prevents the extraction of good‐quality nucleic acids. In this work, we describe a simple approach to divide a single iliac crest bone biopsy into multiple parts. This allows for simultaneous assessments of histology, immunohistochemistry, biomolecular analysis, and tissue banking while preserving the same bone surface area for histomorphometry. © 2020 American Society for Bone and Mineral Research © 2020 The Authors. *JBMR Plus* published by Wiley Periodicals LLC on behalf of American Society for Bone and Mineral Research.

## 1 Introduction

Chronic Kidney Disease‐Mineral and Bone Disorder (CKD‐MBD) is characterized by endocrine disruption of mineral metabolism, bone anomalies, and vascular calcification.^(^ ^1^, ^2^ ^)^ The Kidney Disease: Improving Global Outcomes (KDIGO) 2017 Clinical Practice Guideline Update for the Diagnosis, Evaluation, Prevention, and Treatment of CKD‐MBD recommends that the diagnosis and severity of CKD‐related bone disease be evaluated by iliac crest biopsy if it contributes to treatment changes in patients with CKD G3a to G5D.^(^ ^3^ ^)^

Iliac crest biopsy is now a well‐established diagnostic procedure to describe the histological abnormalities that define high and low bone turnover disease in CKD^(^ ^4^ ^)^ and therefore remains the gold standard for determining bone abnormalities associated with kidney disease.^(^ ^5^ ^)^ Indeed, these histomorphometry parameters provide unique information on bone turnover, mineralization, and volume (TMV) defects to guide the management of renal osteodystrophy and the prevention of CKD‐MBD‐associated fractures.^(^ ^6^, ^7^ ^)^

In standard bone histomorphometry, the whole undecalcified iliac crest biopsy specimen is generally used to obtain quantitative information about static, dynamic, and bone structure parameters by using various tissue colorations. For dynamic parameter analyses, tetracycline, given before the biopsy procedure, is used to assess bone mineralization and formation rate as this compound is deposited within the bone at sites of active mineralization.

Until now, because of the small size and fragility of the iliac crest biopsy specimen, most bone histomorphometrists are reluctant or unable to divide the specimens into segments for further additional analyses such as genetic and molecular analysis. In this work, we show that it is possible to divide the initial iliac crest biopsy specimen into multiple parts while preserving the same bone surface area for histomorphometry analyses, without compromising its integrity. We then show that in addition to tissue banking, other bone segments can be used for immunohistological and molecular biology techniques that are useful for other diagnostic or research purposes.

## 2 Materials and Methods

### 2.1 Subjects, sample handling, and processing

Bone specimens were collected from 31 CKD patients by trans‐iliac crest biopsies. These biopsies were performed in CKD patients with a high risk of fracture or prior fracture in order to guide therapy. All iliac crest biopsies were performed under fluoroscopy by an interventional radiologist, a new procedure that we recently described.^(^ ^8^ ^)^ All procedures were conducted in accordance with the Declaration of Helsinki and were approved by local ethics committee. Written informed consent was obtained for all participants.

**Fig 1:**
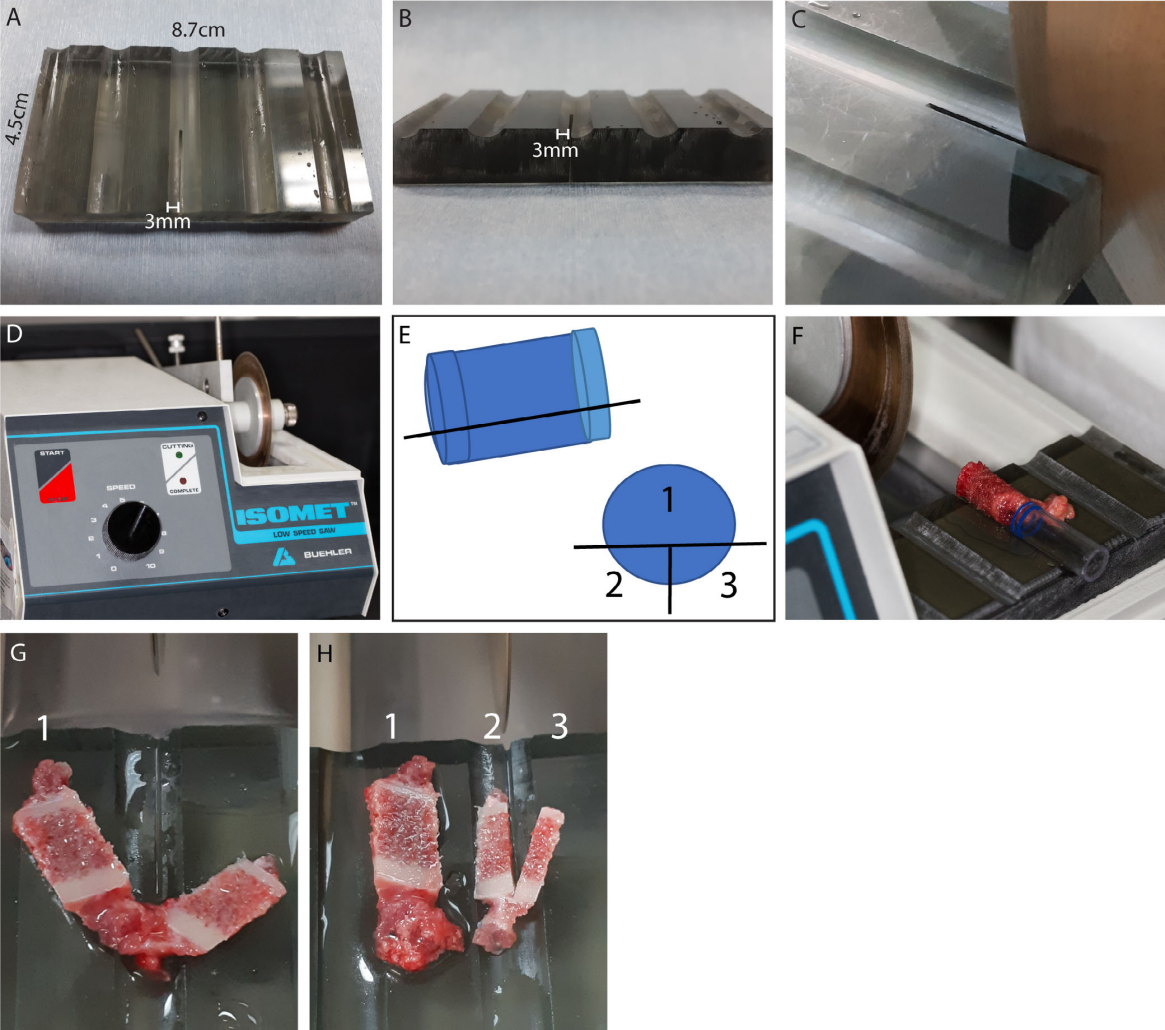
(*A*–*D*) The low‐speed precision saw (Isomet; Buehler, Lake Bluff, IL, USA) equipped with a 4‐inch Isomet Diamond Wafering blade was used to cut the iliac crest biopsy on a custom‐made holding mold. (*E*) Schematic drawing of the bone sectioning. The black lines represent the different cutting parts. (*F*) The biopsy is put on a custom‐made holding mold. (*G*,*H*) The biopsy was first divided into two segments and the smaller segment was further divided in two parts. (*G*,*H*) The largest segment was embedded in MMA for histomorphometry analysis. One of the smaller segments was fixed in 10% buffered formaldehyde for paraffin‐embedding procedure and the other smaller segment was snap‐frozen in liquid nitrogen for mRNA extraction or bio‐banking.

In order to enable dynamic bone measurements, all patients received a course of 3 days of tetracycline (20/mg/kg) 2 times/day at 12 days apart prior to biopsy. The iliac crest biopsy was performed with the Rochester bone biopsy trephine kit (Medical Innovations International, Rochester, MN, USA) to obtain a tissue of 7.5 mm in diameter. At the receipt of the bone biopsy, the bone specimen was put on a custom‐made holding mold (Fig. 1*A‐C*). A low‐speed precision saw equipped with a 4‐inch Isomet Wafering Diamond Blade Fig. 1*D* (Isomet®; Buehler, Lake Bluff, IL, USA), initially designed for cutting different materials with minimal deformation, was used to divide the initial bone specimen into three parts. To minimize the production of dust and particles, we performed wet cuts by placing a receptacle filled with RNase/DNase free water under the blade in the space already designed for this purpose. A first ~1.9‐mm (25%) segment of the biopsy was cut longitudinally (piece 2 and 3 of Fig. 1*E*,*H*). This section was cut in half again. The largest segment (piece 1 of the Fig. 1*E*,*G*,*H*) was fixed in acetone for 24 hours, transferred to ethanol 70% and then embedded in methyl methacrylate (MMA) for histomorphometry analyses. One of the smaller segments was fixed in 10% buffered formaldehyde for 24 hours, decalcified under EDTA, and embedded in paraffin. The other smaller segment was rapidly frozen in liquid nitrogen and stored at –80°C until RNA extraction or bio‐banked for future use.

The custom‐made holding mold was made of 1‐cm‐thick Plexiglas. The Plexiglas was first cut to form a 8.7‐cm × 4.5‐cm rectangle that would perfectly fit into the cutting tray portion of the low‐speed saw. Then a 3‐mm‐deep cavity was drilled on the surface of the Plexiglas, where the biopsies would sit for the cut. Within the cavity, at the edge of the mold toward the blade, we used the low‐speed saw to cut halfway through the Plexiglas, where the biopsy would be cut. This custom‐made mold allowed us to proceed in a similar way for each biopsy.

**Fig 2:**
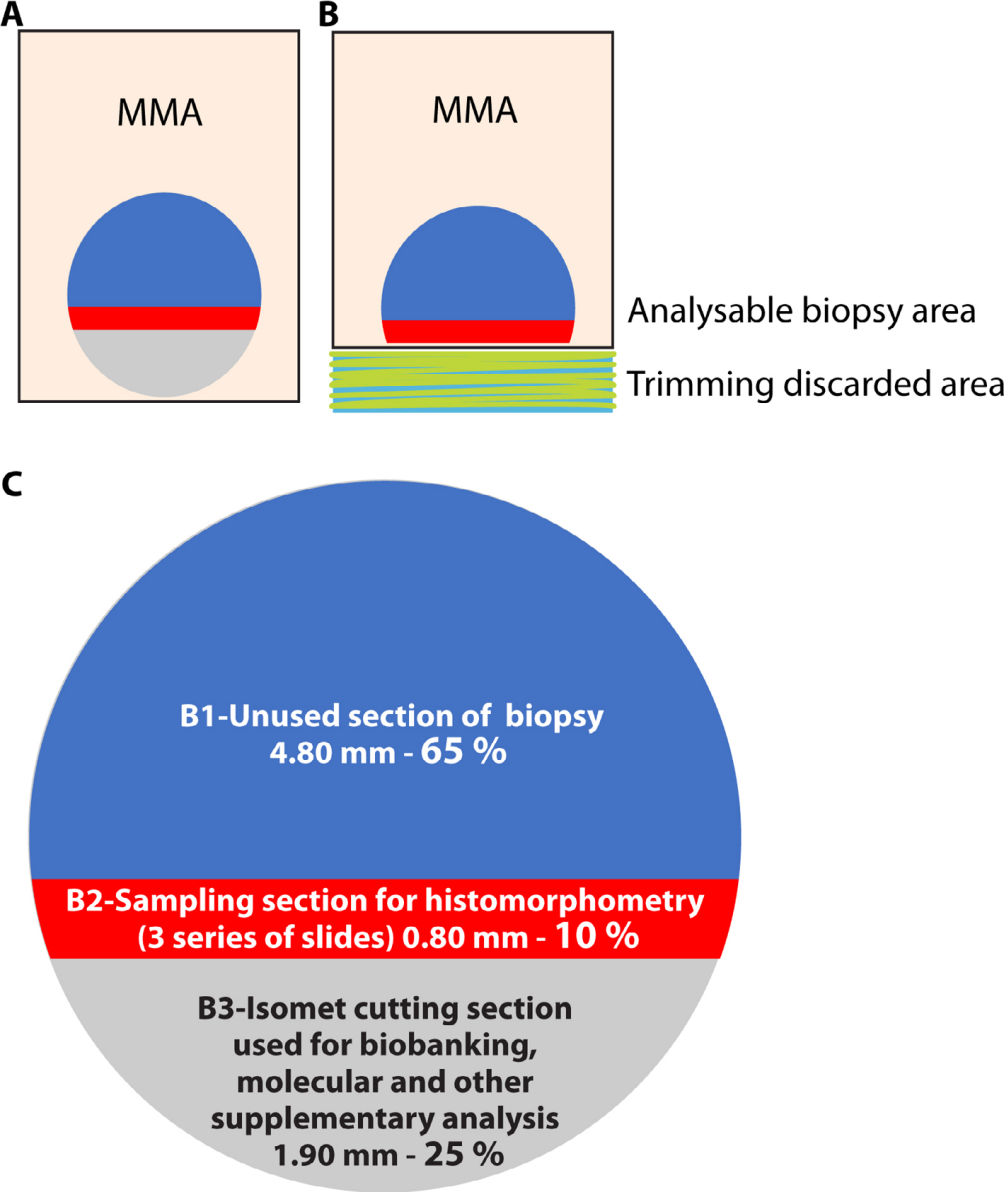
(*A*,*B*) A 2D representation of a 7.5‐mm MMA‐embedded undecalcified bone biopsy for histomorphometry. Traditionally, to reach the optimal analyzable surface (red area), microtome is used for trimming (*B*, green‐yellow hatched area). The trimming residues (corresponding to ~25% of the biopsy) are usually discarded. Our new method proposes to use this 25% of bone tissue (*C*, gray section) for bio‐banking or other purposes, and to use the remaining 75% for histomorphometry analyses. The initial cut takes us directly to the optimal zone of analysis (red section) where we usually use 5‐μm‐thick and 11‐μm‐thick tissue sections at three different levels, separated by a thickness of 250 μm. With this new approach, ~10% or 0.8 mm of the total biopsy (red area) is used for histomorphometry analyses, which is similar to the traditional approach. The rest of the MMA‐embedded biopsy (blue area) can be stored for further analyses.

In Fig. 2A‐C, we illustrate the amount of bone used for standard histomorphometry, which remains similar to the standard approach after the initial low‐speed precision cut. The evaluated outcomes were the integrity of the bone specimens sent for histomorphometry, paraffin, and molecular analyses (pieces 1, 2, and 3 of Fig. 1*H*, respectively).

### 2.2 Histomorphometry

Once embedded in MMA, the larger bone specimen was used for standard histomorphometry analysis. Eight 5‐μm‐thick and one 11‐μm‐thick tissue sections were collected from three different levels, separated by a thickness of 250 μm for each level. Dynamic and static measurements of bone parameters were assessed with unstained (11‐μm‐thick) or stained (5‐μm‐thick) slides with modified Masson‐Goldner's trichrome, respectively. Bone histomorphometry images were captured with a QImaging Exi Aqua camera mounted on an Olympus BX53 microscope (Olympus, Waltham, MA, USA). Images were taken at magnification ×4 and ×20 to evaluate the accuracy and quality of fresh bone sectioning. Bone histomorphometric measurements were analyzed with Bioquant Osteo II (Bioquant Image Analysis Corp., Nashville, TN, USA) in accordance with the standards established by the American Society of Bone and Mineral Research (ASBMR).^(^ ^9^ ^)^

### 2.3 Histology and immunohistochemistry

The following stainings performed on piece 2 of the iliac crest bone specimen were done in order to demonstrate the integrity of the bone tissue and the feasibility of conducting these analyses.

#### 2.3.1 H&E

Five‐μm‐thick slides were deparaffinized in xylene and rehydrated in graded ethanol to distilled water. Slides were then stained with Harris hematoxylin, differentiated in 1% acid alcohol and counterstained with an eosin solution. The staining was performed by the autostainer Sakura Tissue‐Tek Prisma® (Somagen Diagnostics, Edmonton, AB, Canada). Slides were dehydrated in graded alcohol, cleared with toluene, and mounted with Eukitt mounting medium (ESBE Scientific, Markham, ON, Canada).

#### 2.3.2 Tartrate‐resistant acid phosphatase staining

Five‐μm‐thick slides were deparaffinized in xylene and rehydrated in graded ethanol to distilled water. Slides were then put in a Coplin staining jar containing tartrate‐resistant acid phosphatase (TRAP) staining solution at 37°C for 30 min and counterstained with fast green. The slides were dehydrated in graded alcohol, cleared with toluene and mounted with Eukitt mounting medium (ESBE Scientific).

#### 2.3.3 Immunohistochemistry

Five‐μm‐thick slides were deparaffinized in xylene and rehydrated in graded ethanol to distilled water. For antigen retrieval, the slides were put in citrate buffer, preheated at 60°C in a water bath, for 1 hour. After antigen retrieval, endogenous peroxidases were blocked with 3% H_2_O_2_ for 15 min. Nonspecific binding was blocked with 5% normal bovine serum in PBS for 30 min. The slides were then incubated overnight at 4°C with a rabbit anti‐CD68 primary antibody, diluted 1/2500 (specific for macrophage population, including osteoclasts cells) (Sigma‐Aldrich, St. Louis, MO, USA), followed by three washes with PBS, and incubation with secondary antibody (Envision Flex + rabbit linker; DAKO, Carpinteria, CA, USA) at room temperature for 1 hour. Then, a peroxidase polymer (Envision Flex/HRP; DAKO) was added for 30 min before revelation with a solution of DAB (DAKO). Counterstaining with Mayer's hematoxylin was finally performed. The slides were dehydrated in graded alcohol, cleared with toluene, and mounted with Eukitt mounting medium (ESBE Scientific). Histology and immunohistochemistry images were captured with an Infinity 2 camera mounted on an Olympus BX45 microscope (Olympus).

### 2.4 mRNA isolation procedure and qPCR

The whole‐frozen bone segment (piece 3) was used for mRNA extraction. To avoid the thawing of the tissue before the extraction, the bone segment was kept on dry ice. For mRNA extraction, the frozen bone was initially crushed and then grind into a fine powder with a mortar and pestle under liquid nitrogen. Care was undertaken to ensure the presence of liquid nitrogen in the mortar to help with the grinding and minimize RNA degradation. The resulting bone powder was transferred into a tube containing ice‐chilled Trizol reagent (Invitrogen, Carlsbad, CA, USA) and incubated for 15 min. The tubes were then centrifuged at 12,000 rpm (13 000 x g) for 10 min at 4°C. Upon centrifugation, the upper RNA phase was DNase‐treated and cleaned using spin columns (RNeasy Mini kit; QIAGEN, Valencia, CA, USA), according to the manufacturer's protocol. The integrity and the purity of mRNA extracts were analyzed with a Bioanalyzer 2100 (Agilent Technologies, Santa Clara, CA, USA) and a Nanodrop 2000c (Thermo Fisher Scientific, Waltham, MA, USA) on four bone segments. mRNA was then reverse‐transcribed and gene expression of DMP1 (fw‐CGT ATA GAA GGA CCC ACA AAG; rev‐TCT CAG TGT TCC CAG ATA GAT), FGF23 (fw‐CTT GTG CCT CTC CTC TTT ATC; rev‐CCT CTT CCC TAC ACC TTC A), and Osteocalcin (BGLAP, fw‐ACA CTC CTC GCC CTA TT; rev‐CTC CTG CTT GGA CAC AAA) was assessed on a CFX Connect (Bio‐Rad Laboratories, Hercules, CA, USA).

## 3 Results

The described procedure of dividing iliac crest biopsy specimens was performed on 31 patients.

**Fig 3:**
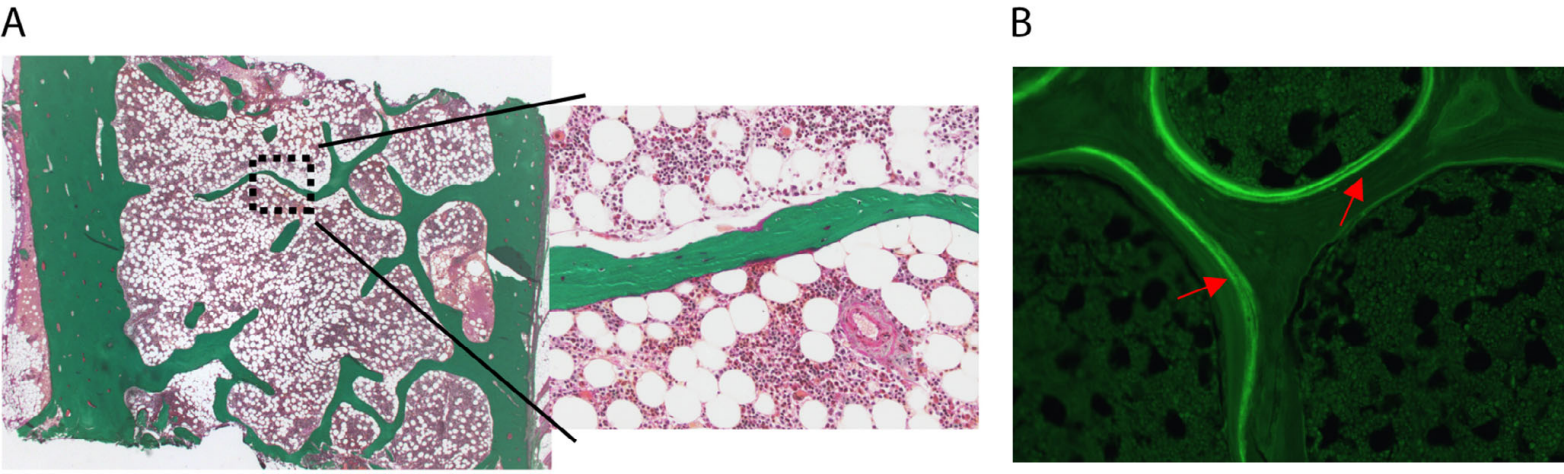
The largest segment of the iliac crest biopsy was embedded in MMA. (*A*) Sections were stained with modified Masson‐Goldner trichrome. (*B*) Unstained tissue sections showed tetracycline labeling (red arrows). All samples were proceeded for standard bone histomorphometry analysis without any further difficulties.

The largest segment embedded in MMA (piece 1) was serially cross‐sectioned and bone slides were obtained for standard bone histomorphometry analysis. Thus, static (bone, trabecular and osteoid surface and volume; osteoblast and osteoclast number; Fig. 3*A*) and dynamic (Fig. 3*B*) parameters were collected according to the ASBMR convention.^(^ ^9^ ^)^ Microarchitectural and cellular integrity, as well as tetracycline labeling of the bone biopsy, were not affected by the initial iliac crest biopsy sectioning. The images shown in this work are representative of each patient's biopsy. The results of histomorphometry analyses for each patient are provided in Supplemental Table 1.

**Fig 4:**
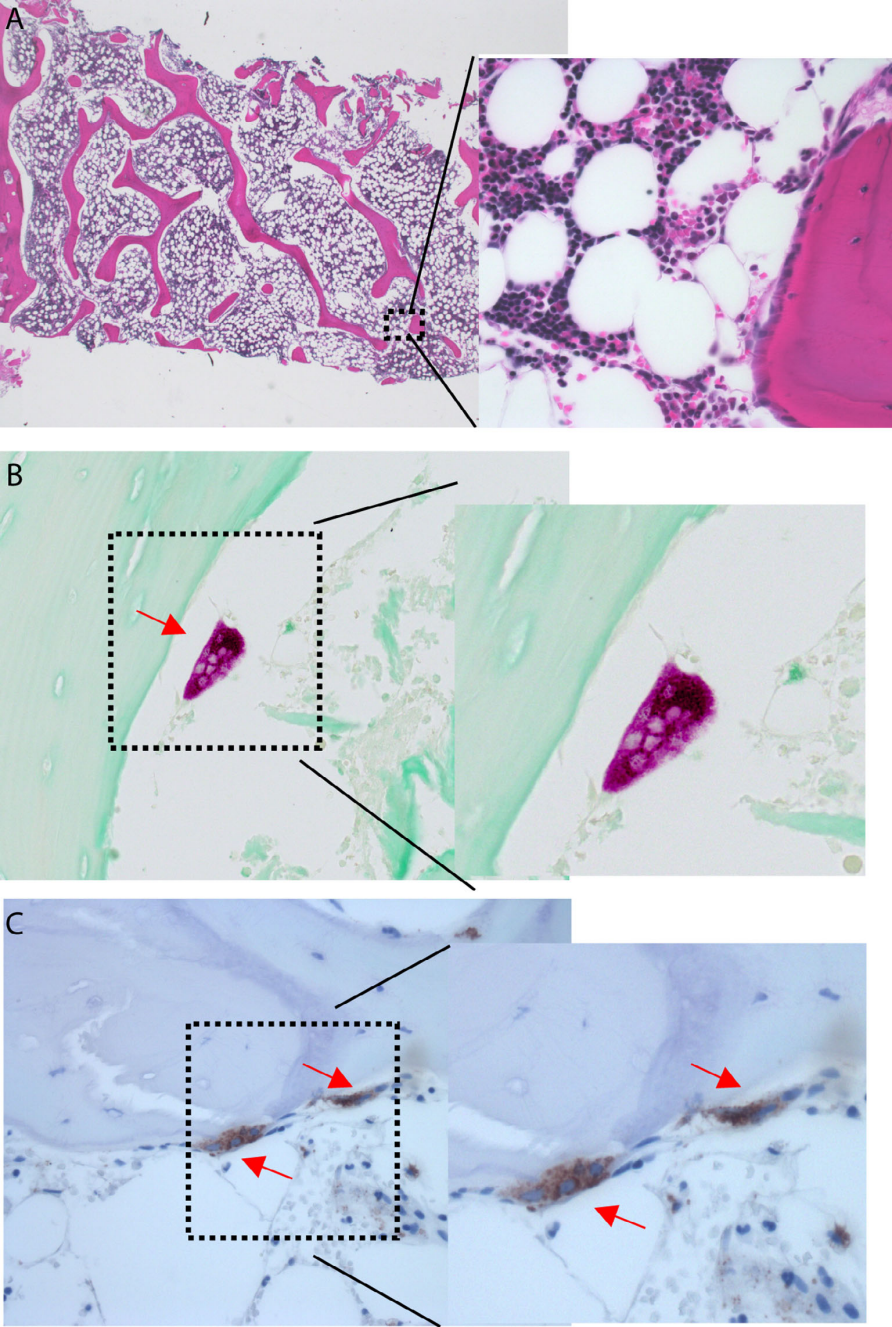
One smaller segment of the iliac crest biopsy was embedded in paraffin. Sections were stained with hematoxylin and eosin (*A*), TRAP (*B*), and immunohistochemistry staining (*C*) were performed for CD68. Specific TRAP and CD68 staining are identified by red arrows.

The paraffin‐embedded segment (piece 2) was serially cross‐sectioned and bone slides were used to perform histology and immunohistochemistry. H&E staining is routinely used in pathology laboratory to study bone marrow structure, cellularity, and assessment of hematological cell lineage. The H&E preparation demonstrated the ability to discriminate hematological bone marrow cellular populations (Fig. 4*A*). TRAP and immunohistochemistry on CD68 were also conducted to demonstrate the feasibility of this technique on the biopsy segment (Fig. 4*B*,*C*).

**Fig 5:**
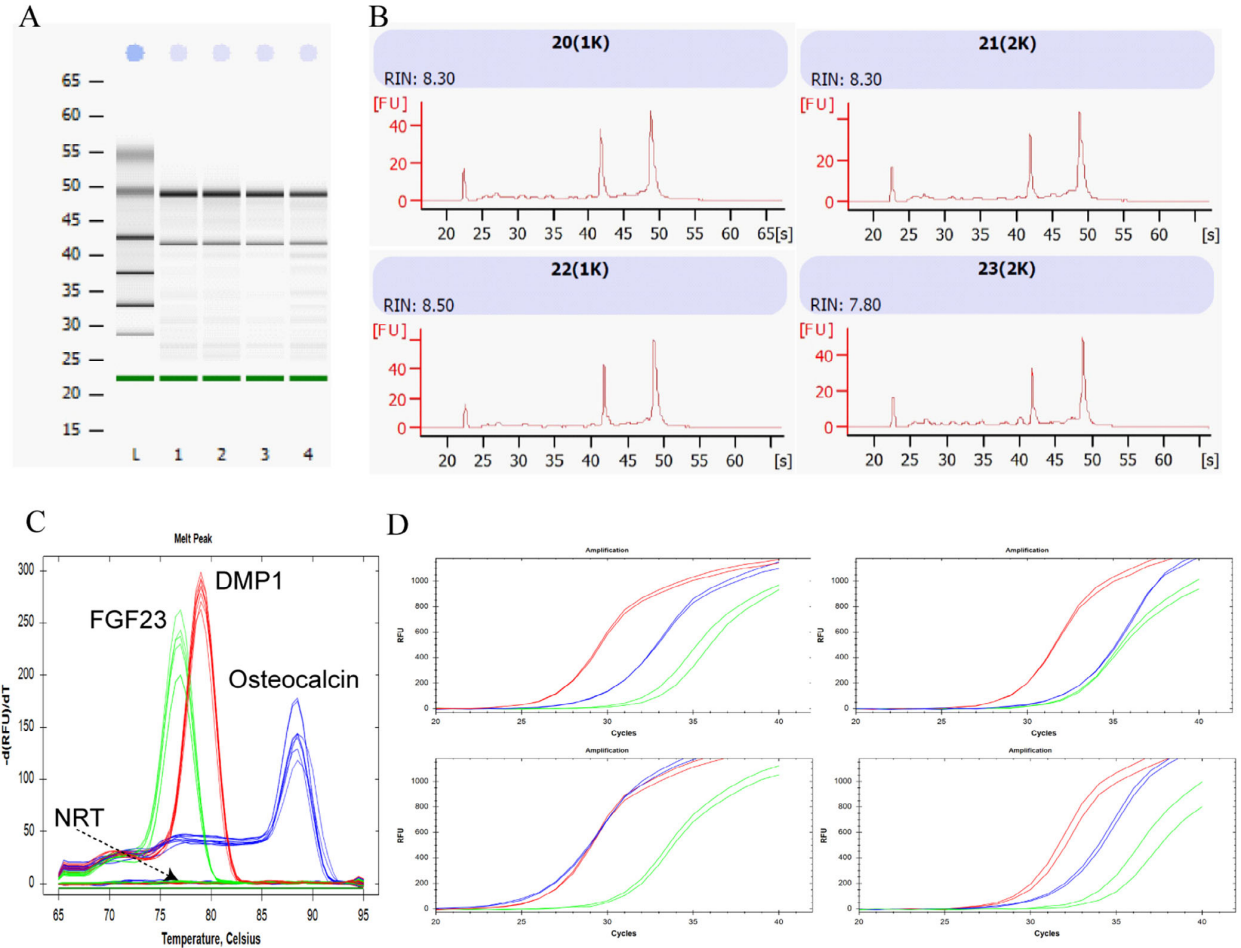
After extraction, mRNAs were analyzed to determine the integrity and concentration with a Bioanalyzer (Agilent). (*A*) Numerical photo of the gel and (*B*) individual assessment of RNA integrity. RIN values were between 7.80 and 8.50. (*C*,*D*) qPCR amplification melt curve and plot for DMP1 (red trace), FGF23 (green trace), and osteocalcin (BGALP, blue trace) gene. All samples showed specific gene amplification. RIN = RNA integrity number.

The last segment (piece 3) was used for mRNA extraction, which allows the study of gene expression. After the cutting of the biopsy, the segment was snap‐frozen in liquid nitrogen and stored until use. Care was taken to proceed as quickly as possible to avoid RNA degradation. A numerical photo of a gel is depicted in Fig. 5*A*. All samples analyzed (*n* = 4) showed 28S and 18S subunit bands. Based on the integrity analysis, the RNA integrity number (RIN) was between 7.6 and 8.5 (Fig. 5*B*). A RIN of 10 is considered as nondegraded RNA. The concentration of mRNA extracted was around 200 ng/μL for each individual in a volume of 50 μL. Spectrophotometry analyses for assessment of RNA purity revealed a 260/280 ratio between 1.94 and 2.00 and a 260/230 ratio between 2.06 and 2.09. RNA values ~2.00 for both ratios are considered free from protein, phenol, or other reagent contaminations. After RNA reverse transcription, qPCR analyses were conducted to validate the presence of osteocyte‐specific and osteoblast‐specific markers (Fig. 5*C*,*D*). All four samples expressed products for DMP1, FGF23, and osteocalcin (BGLAP) genes. Melt curve analyses also showed the specificity of the qPCR products. Only 1 peak per gene amplification products and no NRT (no reverse‐transcriptase ‐negative control for genomic DNA contamination) or unspecific amplification was observed.

Therefore, rapid cutting and processing of the iliac crest biopsy can yield sufficient quantity and quality grade mRNA for gene expression studies.

## 4 Discussion

Iliac crest biopsy for histomorphometric analysis is considered the gold standard to evaluate bone mineralization and turnover, particularly in CKD patients. When an iliac crest biopsy is performed, the whole bone specimen is traditionally embedded in MMA and used for histomorphometry analysis. In this work, we show that it is possible to separate the original biopsy specimen in three segments. Most important, we demonstrate that dividing the biopsy with a low‐speed precision saw does not affect the architecture and integrity of the bone tissues for diagnosis purposes.

At CHU de Québec, l'Hôtel‐Dieu de Québec hospital, we perform iliac crest biopsy in CKD patients for the evaluation of bone turnover and mineralization in order to guide therapy. Using a fluoroscopy‐guided procedure with a standard 7.5‐mm‐diameter trephine (Rochester Kit), we have been able to further refine the bone biopsy technique allowing us to obtain a nearly perfect biopsy specimen in all cases.^(^ ^8^ ^)^ Because standard bone markers are not reliable enough to estimate bone turnover level in CKD, bone histomorphometry analyses thus provide nephrologists essential information on the type of bone disease to guide anti‐fracture therapy.^(^ ^10^, ^11^ ^)^ However, its invasiveness and highly specialized nature restricts its wide utilization.^(^ ^12^ ^)^ Recently, circulating micro‐RNA has been suggested as promising biomarkers of bone turnover level in CKD, but further larger studies are needed to evaluate their potential use in patients.^(^ ^13^ ^)^ We therefore believe that our approach will enable evaluation of standard histomorphometry, while opening the possibility for additional molecular analyses without the necessity to perform a second bone biopsy. Molecular data could then be correlated with standard histomorphometry results to explore new research area in the context of CKD‐related metabolic bone disease.

In our center, this technique allowed us to start a biobank of iliac crest bone tissues in CKD patients for the study of CKD‐MBD. The possibilities for cryopreservation, paraffin embedding, and the preparation of the main specimen for MMA make it possible to maximize the use of the entire biopsy. When the entire bone was used for histomorphometry with the traditional approach, ~25% of the bone was trimmed and discarded to reach the optimal area for analyses. Our technique allows 75% of the initial biopsy specimen to be used for histomorphometry analyses and 25%, which was previously discarded, for other purposes. The largest segment (75%) that is embedded in MMA remains thus similar to the bone surface that would have been used for histomorphometry analyses with the standard approach (without cutting). We believe that a second division of the largest bone tissue segment will even be possible as a recent study confirmed the validity of using half of a standard bone surface embedded in MMA for histomorphometry analysis.^(^ ^14^ ^)^ This will thus allow additional bone segments for further analyses and extra tissue banking. Our new approach is complementary to the standard technique and could be considered for anyone wanting to obtain additional bone tissues from a single iliac crest biopsy.

Although staining and immunohistochemistry procedures on MMA‐embedded tissues have been successfully conducted,^(^ ^15^, ^16^ ^)^ some histological stainings or antibodies may not work well with MMA‐embedded tissues and some centers may be reluctant to use MMA‐embedded specimens. The availability of additional bone tissues will therefore allow to proceed for paraffin embedding whenever needed. Moreover, a major aspect of our cutting technique is the possibility to use a segment of the biopsy for gene expression studies that could be correlated with histomorphometric analyses. Previously, such studies would have required two core biopsies. Indeed, in order to perform both histomorphometry, IHC, western blot and qPCR analyses, Pereira and colleagues^(^ ^17^ ^)^ needed to obtain a second smaller bone specimen with a Jamshidi needle in addition to the standard iliac crest biopsy technique using a Bordier trocart. Here, with a single biopsy specimen, the extracted mRNA concentration is sufficient to perform multiple qPCR analyses. Moreover, it is well known that RNA integrity is a significant variable in the validity of gene expression studies and that poor RNA quality could lead to unreliable data.^(^ ^18^ ^)^ Numerous factors contribute to RNA degradation, such as tissue and environmental RNases, storage conditions, specimen handling, and processing. We have shown that if the bone biopsy specimen is processed in a relatively quick manner, this cutting procedure combined with a rapid snap‐freeze and proper RNA isolation technique of the bone segment will result in the preservation of RNA integrity. However, it is worth mentioning that it would be impossible to discriminate between the cortical, trabecular, and bone marrow compartment so that results should be taken with caution if correlations are to be made with histomorphometric analysis. With our proposed cutting technique, proteins could also be isolated using the protein fraction following mRNA TRIzol extraction,^(^ ^17^ ^)^ although this was not evaluated in this paper.

In conclusion, we showed that the use of a precision cutter to divide a single iliac crest biopsy into multiple segments does not alter the bone surface and microarchitecture of the MMA‐embedded segments used for clinical histomorphometry evaluation. Based on each need and expertise, our simple cutting method allows histomorphometry, immunohistochemistry, molecular biology, bio‐banking, and potential cell culture studies from a single iliac crest biopsy specimen.

## Disclosures

All authors state that they have no conflicts of interest.

## AUTHOR CONTRIBUTIONS

**Sylvain Picard:** Conceptualization; formal analysis; methodology; writing‐original draft; writing‐review and editing. **Christian N. Mayemba:** Formal analysis; methodology; writing‐original draft; writing‐review and editing. **Roth‐Visal Ung:** Formal analysis; methodology; writing‐original draft; writing‐review and editing. **Simon Martel:** Formal analysis; methodology.

5

### PEER REVIEW

The peer review history for this article is available at https://publons.com/publon/10.1002/jbm4.10424.

## Supporting information


**Supplemental Table 1** Histomorphometry parameters for all patients
