# Supplementary material for: Division of an Iliac Crest Bone Biopsy Specimen to Allow Histomorphometry, Immunohistochemical, Molecular Analysis, and Tissue Banking: Technical Aspect and Applications
Source: JBMR Plus. 2020 Oct 29;4(12):e10424. doi: 10.1002/jbm4.10424 (PMC7745880; doi:10.1002/jbm4.10424)
Supplement: Supplementary file 1 — Supplemental Table 1 Histomorphometry parameters for all patients [file JBM4-4-e10424-s001.docx]

| **Patient** | **Age** | **Sex** | **BV/VT** | **N. Oc/E. Pm** | **OV/BV** | **OS/BS** | **Ob.S/BS** | **Medullary**  **Fibrosis** | **Presence of double labeling** | **MS/BS** | **MAR** | **Mlt** | **Aj.AR** | **Remodeling** | **Diagnosis** |
| --- | --- | --- | --- | --- | --- | --- | --- | --- | --- | --- | --- | --- | --- | --- | --- |
|  |  |  | **(%)** | **(mm)** | **(%)** |  |  |  |  |  | **(μm/d)** | **(d)** | **(μm/d)** |  |  |
| **1** | 58 | F | 19,40 | 5,70 | 10,10 | 0,570 | 0,064 | ++ | Yes | 0,196 | 0,62 | 107,5 | 0,10 | Low | Osteomalacia |
| **2** | 61 | F | 8,50 | 6,00 | 10,10 | 0,514 | 0,064 | ++ | Yes | 0,088 | 0,60 | 83,6 | 0,10 | Normal | Mineralization defects/ light HPT |
| **3** | 63 | M | 13,00 | 2,40 | 2,70 | 0,160 | 0,004 | 0 | rare |  | 0,52 |  |  | Low | Low turnover bone disease |
| **4** | 50 | F | 10,30 | 2,40 | 4,10 | 0,263 | 0,023 | + | rare |  | 0,65 |  |  | High | HPT |
| **5** | 58 | F | 14,80 | 4,10 | 1,50 | 0,107 | 0,015 | + | Yes | 0,066 | 0,59 | 18,7 | 0,36 | Normal | Low turnover bone disease |
| **6** | 28 | F | 14,80 | 4,20 | 0,20 | 0,017 | 0,000 | 0 | Not observed |  |  |  |  | Low | Low turnover bone disease |
| **7** | 65 | F | 9,30 | 2,80 | 2,30 | 0,118 | 0,010 | 0 | rare |  | 0,49 |  |  | Low | Low turnover bone disease |
| **8** | 78 | F | 9,90 | 5,30 | 8,50 | 0,368 | 0,023 | ++ | Not observed |  |  |  |  | High | Mixed osteodystrophy |
| **9** | 45 | F | 19,60 | 2,50 | 1,70 | 0,103 | 0,011 | ++ | Not observed |  |  |  |  | Normal | Low turnover bone disease |
| **10** | 65 | M | 9,20 | 1,30 | 0,80 | 0,061 | 0,200 | 0 | rare |  | 0,52 |  |  | Low | Low turnover bone disease |
| **11** | 75 | F | 15,90 | 4,20 | 6,70 | 0,389 | 0,003 | + | Yes |  | 0,48 |  |  | Low | Relative low turnover bone disease |
| **12** | 63 | F | 23,30 | 4,10 | 0,30 | 0,024 | 0,001 | + | rare |  | 0,54 |  |  | Low | Low turnover bone disease |
| **13** | 37 | M | 16,00 | 4,70 | 7,30 | 0,372 | 0,046 | +++ | Yes | 0,424 | 1,06 | 7,87 | 1,22 | High | HPT |
| **14** | 68 | M | 17,70 | 6,10 | 3,50 | 0,208 | 0,012 | + | Yes | 0,105 | 0,71 | 20,7 | 0,36 | High | HPT |
| **15** | 56 | F | 20,10 | 7,90 | 2,10 | 0,139 | 0,015 | + | Yes |  | 0,71 |  |  | Low | Low turnover bone disease |
| **16** | 78 | F | 10,40 | 7,30 | 3,20 | 0,155 | 0,005 | + | rare |  | 0,55 |  |  | Low | Relative low turnover bone disease |
| **17** | 54 | M | 21,10 | 5,30 | 10,90 | 0,446 | 0,040 | ++ | Yes |  | 0,71 |  |  | High | Mixed osteodystrophy |
| **18** | 67 | F | 9,30 | 5,20 | 0,02 | 0,002 | 0,000 | 0 | Not observed |  |  |  |  | Low | Low turnover bone disease |
| **19** | 52 | F | 22,60 | 4,90 | 0,86 | 0,088 | 0,013 | ++ | Yes | 0,028 | 0,96 | 18,9 | 0,36 | Normal | Normal remodeling |
| **20** | 61 | F | 16,90 | 5,80 | 3,20 | 0,216 | 0,026 | + | Yes | 0,076 | 0,78 | 23,6 | 0,27 | Normal | Normal remodeling |
| **21** | 71 | F | 22,80 | 2,21 | 0,33 | 0,024 | 0,001 | + | Yes |  | 0,44 |  |  | Low | Relative low turnover bone disease |
| **22** | 55 | F | 19,70 | 3,70 | 2,90 | 0,248 | 0,021 | ++ | Yes | 0,222 | 0,57 | 13,0 | 0,51 | High | Light HPT |
| **23** | 41 | F | 26,50 | 4,30 | 3,00 | 0,160 | 0,018 | ++ | Yes | 0,103 | 0,69 | 17,9 | 0,44 | Normal | Normal remodeling |
| **24** | 78 | F | 19,40 | 2,50 | 0,91 | 0,052 | 0,003 | + | Yes |  | 0,48 |  |  | Low | Relative low turnover bone disease |
| **25** | 74 | M | 9,50 | 0,00 | 1,10 | 0,088 | 0,000 | 0 | Not observed |  |  |  |  | Low | Low turnover bone disease |
| **26** | 57 | F | 8,30 | 3,60 | 4,60 | 0,275 | 0,039 | 0 | Yes | 0,116 | 0,39 | 38,1 | 0,16 | Normal | Osteoblastic osteoporosis |
| **27** | 78 | M | 12,70 | 1,40 | 1,30 | 0,079 | 0,001 | + | rare | 0,017 | 0,43 | 64,9 | 0,09 | Low | Low turnover bone disease |
| **28** | 67 | M | 15,10 | 3,00 | 1,20 | 0,071 | 0,002 | ++++ | rare |  | 0,67 |  |  | High | HPT |
| **29** | 84 | M | 5,04 | 0,00 | 0,70 | 0,040 | 0,001 | 0 | Not observed |  |  |  |  | Low | Low turnover bone disease |
| **30** | 72 | F | 6,90 | 4,20 | 3,70 | 0,225 | 0,010 | + | Yes | 0,098 | 0,63 | 24,0 | 0,28 | Normal | Normal remodeling |
| **31** | 64 | M | Low | 1,50 | 2,50 | 0,176 | 0,000 | + | rare |  | 0,37 |  |  | Low | Low turnover bone disease |

Supplemental Table 1. Histomorphometry parameters for all patients

From Dr Louis-Georges Ste-Marie and Natalie Dion, CHUM-Department of Pathology, Montreal, Canada; M: Male; F: Female; HPT: Hyperparathyroidism bone disease.
